# Transient Neonatal Diabetes Mellitus with the Rare Association of Nonsuppurative Sialadenitis and Genetic Defects in 6q24

**DOI:** 10.1155/2021/5901898

**Published:** 2021-08-11

**Authors:** Manal Mustafa, Nitin Ramdas, Mahmoud Elhalik, Arif Faquih

**Affiliations:** ^1^Pediatric Endocrinology Division, Department of Pediatrics, Latifa Women and Children Hospital, P.O. Box 9115, Dubai, UAE; ^2^Neonatal Intensive Care Unit, Latifa Women and Children Hospital, P.O. Box 9115, Dubai, UAE

## Abstract

**Background:**

Transient neonatal diabetes mellitus (TNDM) is the most common cause of diabetes in the first week of life, with an overall incidence of 1 in 90,000 to 160,000 live births. TNDM occurs soon after birth and undergoes spontaneous remission during infancy; however, it may relapse to a permanent form of diabetes mellitus in childhood or adolescence. We report a case of TNDM due to hypomethylation on chromosome 6q24, associated with a rare clinical finding of nonsuppurative submandibular sialadenitis managed by subcutaneous insulin, and who underwent remission by three months of age. *Case Presentation*. We report a male neonate of Arab ancestry delivered by caesarean section at 37 weeks of gestation. He had intrauterine growth retardation with a birth weight of 2.099 kg. He presented with hyperglycemia on the first day of life, which was managed with parenteral insulin infusion. Blood glucose control was initially difficult to achieve due to difficulties in preparing such small doses of insulin and the significant variations in blood glucose concentrations, without ketosis. Blood tests revealed low serum insulin and C-peptide levels. Genetic analysis revealed multiple loci hypomethylation of the *PLAGL1/HYMAI-DMR* in the TNDM region in chromosome 6q24 and two pathogenic heterozygous variants in the *ZFP57* gene. Segregation analysis showed that both parents were heterozygous carriers of familial *ZFP57* variants. The clinical course was associated with bilateral nonsuppurative sialadenitis, which is extremely rare among newborns.

**Conclusion:**

Sialadenitis is a well-known phenomenon that is rarely diagnosed in neonates. To the best of our knowledge, this is the first case report to describe the exceedingly rare association of nonsuppurative submandibular sialadenitis in a neonate with TNDM due to multiple loci hypomethylation of the *PLAGL1/HYMAI-DMR* in the TNDM region in 6q24 and heterozygous pathogenic variants in the *ZFP57* gene.

## 1. Introduction

Transient neonatal diabetes mellitus (TNDM) is the most common cause of diabetes in the first week of life, with an overall incidence of 1 in 90,000 to 160,000 live births [1, 2]. TNDM occurs soon after birth and undergoes spontaneous remission during infancy; however, it may relapse to a permanent form of diabetes mellitus (DM) in childhood or adolescence. This condition can be sporadic or inherited, and the majority of the cases are due to defects at the TNDM locus on chromosome 6q24.

Here, we report a case of TNDM due to hypomethylation on chromosome 6q24, associated with the rare clinical finding of nonsuppurative submandibular sialadenitis managed by subcutaneous insulin, wherein the patient underwent remission by three months of age. Informed consent was taken from the patient's parents for publication of the findings.

## 2. Case Presentation

Our patient was a male baby born to second-degree consanguineous parents at 37 weeks of gestation with intrauterine growth restriction (IUGR) revealed by antenatal ultrasound (USS), as well as hyperglycemia. The mother was 29 years old, G6P4 + 1, with gestational diabetes (diet-controlled) and pregnancy-induced hypertension. She also had the thalassemia trait. There was no family history of diabetes, and the other four siblings of the baby were healthy. The baby was born by emergency lower segment caesarean section at a peripheral hospital due to fetal distress, with Apgar scores of 8 and 9 at 1 and 5 min, respectively. His first blood sugar level checked after birth was high, at 304 mg/dL (16.9 mmol/L). Hence, the baby was referred to our tertiary neonatal intensive care unit for the management of hyperglycemia.

His initial clinical examination was normal, except for macroglossia and low birth weight. His growth parameters were as follows: birth weight, 2099 g (12th centile “−2.2SD”); length, 45 cm (3rd centile “−1.8SD”); and head circumference, 33 cm (12th centile “−1.3SD”). He was started on oral feeds and underwent routine blood glucose monitoring for the first 24 hours, which showed hyperglycemia ranging from 200 to 300 mg/dL. He was kept nil by mouth because of vomiting episodes, with close monitoring of blood glucose levels. Other possible causes of neonatal hyperglycemia, such as sepsis and stress, were excluded. Continuous intravenous insulin infusion was started given the persistent hyperglycemia (range, 300–600 mg/dL) after collecting the relevant blood test results. Initial investigations revealed glycosuria (4+) and ketonuria (1+), low serum insulin (<0.4 Uiu/mL (2.6–24.9 Uiu/mL)), and low serum C-peptide level (0.11 nmol/L (0.37–1.47 nmol/L) when serum glucose was 446 mg/dL (24.8 mmol/L). HbA1c was 3.5%. Intravenous insulin infusion (Humulin R) was started at a rate of 0.08 u/kg/hour during admission. The intravenous insulin infusion rate was adjusted to maintain blood glucose <200 mg/dL (11.1 mmol/L). Abdominal USS revealed a normal pancreas and kidneys. The baby was started on oral feeds on day 3, which was gradually increased to full oral feeds at 150 mL/kg/day with acceptable blood glucose levels <250 mg/dL (13.9 mmol/l).

On day 8 of life, after establishing full oral feeds, insulin infusion was stopped and subcutaneous multiple daily insulin injections were started in the form of short-acting insulin (lispro; an initial dose of 0.025 units PRN, if BG > 250 mg/dL (13.9 mmol/L)) and long-acting insulin (detemir; 0.25 units SC daily (0.125 *μ*/kg/day)). To prepare these exceedingly small doses of insulin, we diluted insulin in normal saline and used a 30-unit (0.30 mL) 31 g BD insulin syringe. For example, to prepare 0.025 units of the insulin lispro, we used a 30-unit BD insulin syringe (0.30 mL) and diluted 1 unit of lispro with 19 units of 0.9% saline, making up a total volume of 20 units. Hence, 2.5 units of this mixture contain 1 unit of lispro. Similarly, to prepare 0.25 units of lispro, we used a 30-unit BD insulin syringe (0.30 mL) and diluted 1 unit of lispro with nine units of 0.9% saline, making up a total volume of 10 units. Hence, 2.5 units of this mixture contain 1 unit of lispro.

Repeated blood investigations during the episode of hyperglycemia (323 mg/dL) revealed low serum insulin (1.4 Uiu/mL) and low C-peptide level (0.18 nmol/L). Molecular genetic analysis for NDM was performed to delineate the underlying genetic causes. However, the newborn screening results were negative.

**Figure 1:**
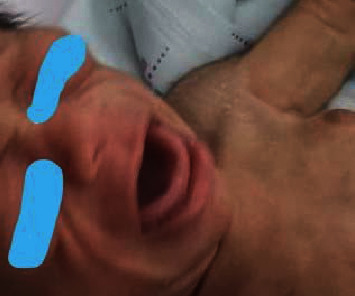
Macroglossia.

**Figure 2:**
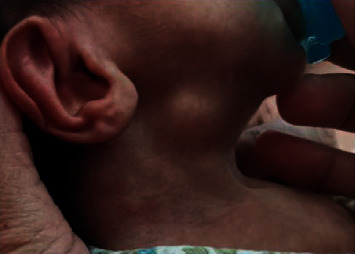
Submandibular gland enlargement.

**Figure 3:**
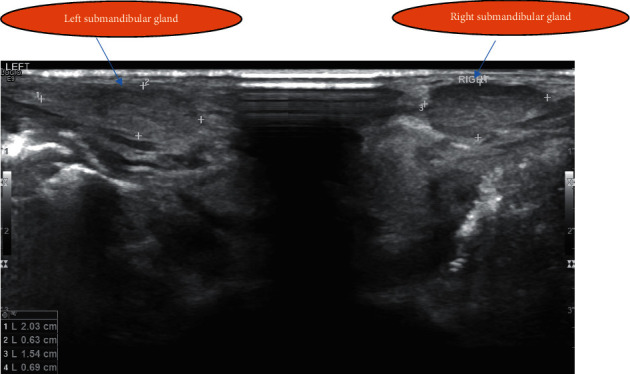
Ultrasound with bilateral submandibular gland enlargement (hypoechoic area).

On day 13 of life, due to early morning hyperglycemia episodes, detemir was changed to glargine because of its prolonged action, with a dose of 0.15 units (0.075 *μ*/kg/day) SC. Blood glucose monitoring prefeeds and three hourly feeds were continued. The glargine dose was increased to 0.4 units daily (0.2 *μ*/kg/day) due to persistent hyperglycemia. On day 17 of life, due to persistent prefeed nocturnal hyperglycemia (250–400 mg/dL), glargine was reverted to detemir, at a dose of 0.4 units (0.2 *μ*/kg/day) SC once daily. The detemir dose was administered twice daily on the next day for further control of hyperglycemia (range, 350–500 mg/dL). The insulin doses were frequently increased and adjusted according to the blood sugar readings and feeding. Blood glucose control had been initially challenging due to the difficulties in preparing small amounts of insulin and the significant variations in blood glucose concentrations, without ketosis. The baby was regularly checked by a dietician to optimize calorie intake and ensure normal growth. During the NICU course, on day 13 of life, routine daily examination showed bilateral submandibular nontender swelling in addition to macroglossia (Figures 1 and 2). Neck USS (Figure 3) showed bilateral submandibular gland swelling. The right and left sides measured 1.5 × 0.7 cm and 2 × 0.6 cm, respectively. No lymph nodes were observed. Subsequently, on day 23, the submandibular gland swelling subsided without any clinical concerns. All possible aetiologies leading to submandibular sialadenitis were excluded, including a negative sepsis screen. In our reported patient, the submandibular gland swelling was nonsuppurative and spontaneously resolved by the age of 4 weeks.

On day 24, routine clinical examination revealed bilateral hypertrophy at the insulin injection sites on the anterior thighs, with hyperglycemia episodes. Hence, we changed the injection sites to the upper limbs and lateral thighs. We aimed to ensure proper insulin dilution and insulin injection throughout the patient's hospital stay. Blood glucose levels were stabilized using long-acting insulin (detemir) of 0.25 units SC twice daily (0.25 *μ*/kg/day) and PRN short-acting insulin lispro (If BG > 200 mg/dL (11.1 mmol/L)). The baby showed adequate weight gain with on-demand feeds of expressed breast milk and formula milk feeds.

The genetic study showed abnormal methylation of the TNDM critical region in chromosome 6q24, as shown in Table 1.

## 3. Method for Gene Analysis

The coding exons of known genes for neonatal diabetes mellitus were enriched using Roche/N sequence capture technology and sequenced on an Illumina system (next-generation sequencing, NGS). Additionally, analysis of the methylation status of the PLAGL1/HYMAI-DMR (DMR: differentially methylated region) in 6q24 as well as deletion/duplication analysis of the TNDM critical region 6q24 was performed by applying methylation-sensitive MLPA (multiplex ligation-dependent probe amplification; SALSA MLPA kit ME033-A1 TNDM).

  (Analyzed genes: *ABCC8*, *AGPAT2*, *BSCL2*, *CISD2*, *EIF2AK3*, *EIF2S3*, *FOXP3*, *GATA4*, *GATA6*, *GCK*, *GLIS3*, *HNF1A*, *HNF1B*, *HNF4A*, *IER3IP1*, *ILR2RA*, *INS*, *KCNJ11*, *KLF11*, *LRBA*, *MNX1*, *MTTL1*, *NEUROD1*, *NEUROG3*, *NKX22*, *PAX6*, *PDX2*, *PLAGL1*, *PTF1A*, *RFX6*, *SLC19A2*, *SLC29A3*, *SLC29A3*, *SLC2A2*, *STAT3*, *WFS1*, *ZFP57*)

## 4. Interpretation of Genetic Study

The copy number analysis of the TNDM critical region by MLPA revealed no indication of a deletion or duplication in the analyzed areas. However, the TNDM critical region's methylation in chromosome 6q24 by MLPA showed a loss of signal intensity for the three methylation-sensitive MLPA probes of *PLAGL1/HYMAI-DMR* (18458-L25628, 18460-L26610, and 15755-L25779). This finding most likely corresponds to the hypomethylation of *PLAGL1/HYMAI-DMR*. The results were confirmed by performing an independent MLPA analysis.

Additionally, NGS analysis identified the heterozygous deletion of one base in exon 4 of the *ZFP57* gene (c. 1024delC), leading to frameshift (P. (Gln342Argfs*∗*13)), resulting in a premature stop codon and subsequent mRNA degradation (nonsense-mediated decay) or protein truncation. This variant has already been described in a homozygous state in a patient with a 6q24 methylation defect. However, the allele frequency of this variant in the general population has not yet been documented. The variant was also classified as pathogenic.

To confirm compound heterozygosity of the detected *ZFP57* variants, segregation analysis of the parents was performed, which showed that both parents were heterozygous carriers of the familial *ZFP57* variants (the father was a heterozygous carrier of the c.1383delC P.(Tyr462llefs*∗*16) variant, and the mother was a heterozygous carrier of the c.1024delC P. (Gln342Argfs*∗*13) variant).

The patient was discharged from the hospital at the age of 1 month in good general condition, with a body weight of 2.850 kg (0.5th centile, −2.5SD) and a normal neurological examination and brain ultrasound. During follow-up in the pediatric diabetes clinic, the detemir dose was reduced gradually based on the patient's normal blood sugar and was subsequently stopped at two months of age due to the absence of hyperglycemic episodes. The follow-up HbA1c at the age of 3 months was 3.8%.

Currently, the baby is six months old and has not been administered insulin for the last three months. He is thriving well with normal random blood sugar levels, and his HbA1c level at the age of 5 months was 4.1%.

## 5. Discussion

This case report describes a male neonate with TNDM due to multiple loci hypomethylation of the *PLAGL1/HYMAI-DMR* in the TNDM region in 6q24, as well as two pathogenic heterozygous variants in the *ZFP57* gene. Glycemic control had been quite challenging, requiring multiple-dose adjustments to prepare small doses of insulin due to significant variations in the patient's blood glucose concentrations.

Neonates affected with TNDM are diagnosed at a younger age, with significant IUGR, requiring less insulin for glucose control and presenting with less severe ketosis compared to PNDM. Permanent NDM (PNDM) occurs during the first six months of life and does not go into remission [3].

The incidence of NDM in the Middle East region is among the highest in the world due to high consanguineous marriage rates, and its spectrum is different from that in Europe and the USA. NDM data from the Gulf region are limited to a few studies on PNDM [4]. In a cohort study conducted by Deeb et al., the estimated incidence of NDM in Abu Dhabi, the capital of the United Arab Emirates, was 1 in 29,241 live births. In this study, 23/25 had PNDM (incidence 1 : 31,900), whereas 2/25 had TNDM (incidence 1 : 350,903) [5]. A Turkish study by Demirbilek et al. found that the overall annual incidence of NDM (and PNDM) in the southeastern Anatolian region of Turkey was at least one in 30,000 live births (PNDM: one in 48,000 live births). The incidence of TNDM caused by mutation of the *ZFP57* gene was also high in this Turkish cohort, where 2/3 TNDM cases with chromosome 6q24 methylation abnormalities had *ZFP57* gene mutations. The prevalence of *ZFP57* gene mutations correlated with that of consanguinity; hence, TNDM can be suspected because the recurrence risk in affected pedigrees is 25% [6]. To determine the true incidence rate of NDM, multicenter and larger nationwide studies are necessary.

Twenty percent of patients with NDM will ultimately have TNDM resolved in infancy (usually by 13 to 18 weeks of age); however, this may recur later in life [7–9]. The underlying genetic defects of TNDM include the following:

- Overexpression of the imprinted region of chromosome 6q24 includes the genes *PLAGL*, *HYMAI*, and *ZPF57* [10]. The overexpression of this region is attributed to a mutation in the zinc finger transcription factor *ZPF57*, leading to hypomethylation of the imprinted loci. It could also be due to the duplication of 6q24 from the paternal uniparental disomy or an unbalanced duplication of the paternal chromosome 6 [10].
- Activating mutations of *KCNJ11* or *ABCC8* that encode subunits of the K-ATP channel can lead to both transient and permanent NDM. For patients with these gene mutations, the need for therapeutic interventions determines whether the DM is transient or permanent.
- Autosomal recessive mutations of the *INS* gene encode preproinsulin, as reported in some cases. These mutations act by reducing preproinsulin peptide synthesis. Garin et al. reported that TNDM is usually observed in patients with recessive mutations (26%, *P*=0.001). Patients with NDM resulting from recessive *INS* mutations had a markedly different phenotype, with lower birth weight (median SD score −3.2 vs. −2.0, *P* < 0.001) and an earlier age of diagnosis (median age in weeks 1 [0, 3] vs. 10 [5, 11], *P* < 0.001) [12].

A cohort study conducted by Shield et al. in infants with TNDM showed that the median age at presentation was three days of life, the majority were born small for their gestational age, and the median age of insulin therapy was 12 weeks [13]. Hyperglycemia due to TNDM is markedly severe and is usually accompanied by low or undetectable insulin and C-peptide levels. Hyperglycemia can rarely progress to ketoacidosis, but more so in PNDM than in TNDM. IUGR commonly affects >95% of patients with TNDM, typically developing in the third trimester. A 2002 French cohort study found that TNDM is associated with more IUGR (74% vs. 36%) and early diagnosis (median age, six days; range, 1–81 days vs. median age, 27 days; range, 1–127 days) than PNDM. Patients with TNDM with chromosome 6q abnormalities have a significantly lower birth weight than those with K-ATP channel mutations [14].

Docherty et al. evaluated the genotype-phenotype correlation in an international cohort of patients with TNDM. They reported that the most common congenital abnormalities were macroglossia and umbilical hernia (44% and 21%, respectively). Less frequently reported congenital abnormalities included dysmorphic facial appearance (18%), renal tract abnormalities (duplex kidneys, hydronephrosis, dilated renal pelvis, and vesicoureteral reflux) (9%), cardiac anomalies (ductus arteriosus, tetralogy of Fallot, atrial-septal defects, and persistent foramen ovale) (9%), clinodactyly, polydactyly, and nail and short finger abnormalities (8%), and hypothyroidism (4%) [15]. Our reported patient with TNDM has an additional finding of nonsuppurative submandibular salivary gland sialadenitis. Inflammation of the salivary gland is an uncommon condition in the neonatal period, with a paucity of data on predisposing factors of this entity, and the cause remaining unclear in most cases. Possible causes reported among newborns include dehydration, calculi, prolonged orogastric feeding practices, duct stricture, anatomic deformities of the oral cavity, congenital oral cysts, and sialectasis [16, 17].

Isolated submandibular acute inflammatory sialadenitis is a rare phenomenon in the neonatal period. One case report in an adult population described sialadenitis associated with DM [18]. Acute inflammatory submandibular sialadenitis associated with prematurity and maternal drug use has also been reported [19]. Neonatal suppurative submandibular sialadenitis caused by infectious agents such as *Staphylococcus aureus*, *Pseudomonas aeruginosa*, *Escherichia coli*, and *Moraxella catarrhalis* has also been reported [20]. In the present case report, we described the exceedingly rare association of nonsuppurative submandibular sialadenitis in a neonate with TNDM due to multiple loci hypomethylation of the *PLAGL1/HYMAI-DMR* in the TNDM region in chromosome 6q24 and the heterozygous pathogenic variants in the *ZFP57* gene. It is unclear if the presence of nonsupportive sialadenitis in this patient has a cause or was merely coincidental.

As lower HbA1c level (high HbF) is observed in relation to plasma glucose level, it cannot be used as a glycemic control indicator in NDM. Glycated albumin, unaffected by HbF, can be used as a glycemic control indicator in NDM [21, 22].

In most cases, TNDM is associated with anomalies in chromosome 6q24 (70%). The underlying genetic defects included overexpression of the imprinted region of chromosome 6q24, including the genes *PLAGL, HYMAI*, and *ZPF57* [11, 23]. Overexpression of this region has been attributed to a mutation in the zinc finger transcription factor *ZPF57*, leading to hypomethylation of the imprinted loci or duplication of chromosome 6q24 from either paternal uniparental disomy or an unbalanced duplication of the paternal chromosome 6. In a proportion of patients, hypomethylation appears to be purely epigenetic, without any detectable underlying genetic cause, and exclusively affects the *DMR* in TNDM. Another report by Mackay et al. concluded that hypomethylation of multiple imprinted loci (HIL) is observed, with a portion of these cases associated with genetic mutations in the *ZFP57* gene [24]. Mutations in the *ZFP57* gene are associated with an autosomal recessive imprinting disorder with inherently variable epigenetic effects and variable clinical features. *ZFP57* gene mutations are associated with the first heritable global imprinting disorder described in humans, which is compatible with life [25].

Clinical course was complicated by injection site hypertrophy, needing change in insulin injection site until subcutaneous fat development.

In conclusion, although sialadenitis is a well-known phenomenon, it has rarely been diagnosed in neonates. To the best of our knowledge, this is the first case report to describe the exceedingly rare association of nonsuppurative submandibular sialadenitis in a neonate with TNDM due to multiple loci hypomethylation of the *PLAGL1/HYMAI-DMR* in the TNDM region in 6q24 and the heterozygous pathogenic variants in the *ZFP57* gene. In our reported case, the submandibular gland swelling was nonsuppurative and spontaneously resolved by the age of 4 weeks.

## Figures and Tables

**Table 1:** Molecular genetic analysis for neonatal diabetes mellitus.

Gene (isoform)	Phenotype MIM number	Variant	Zygosity	MAF genome AD (%)	Classification
*ZPF57* (NM_001109809.2)	601410 (AD)	c.1024delC	Heterozygous	0	Pathogenic
		P. (Gln342Argfs*∗*13)			
		Chr6:29640863			
		c.1383delC	Heterozygous	0	Pathogenic
		P. (Tyr462llefs*∗*16)			
		chr6:29640504			

## Data Availability

The data used to support the findings of this study are available from the corresponding author upon request.

## Abbreviations

IUGR: Intrauterine growth restriction
USS: Ultrasound
SC: Subcutaneous
NDM: Neonatal diabetes mellitus
TNDM: Transient neonatal diabetes mellitus
PNDM: Permanent neonatal diabetes mellitus
BD: Twice daily
BG: Blood glucose
PRN: “pro re nata” as needed
EBM: Expressed breast milk.
